# Do Patient-Reported Quality-of-Life (QoL) Scales Provide an Adequate Assessment of Patients with Cryptoglandular Anal Fistulae? A Systematic Review of Measurement Instruments and Their Content Validity

**DOI:** 10.3390/clinpract12040066

**Published:** 2022-08-15

**Authors:** Nusrat Iqbal, Rishi Shah, Laith Alrubaiy, Phil Tozer

**Affiliations:** Robin Phillips’ Fistula Research Unit, St Mark’s Hospital, London HA1 3UJ, UK

**Keywords:** quality of life, QoL, outcome measures, anal fistula, COSMIN, patient-reported outcome measures, PROMs

## Abstract

Background: Cryptoglandular anal fistulae can significantly affect patient quality of life (QoL), making it essential to ensure that any study of fistula treatment assesses the impact on QoL. The aim of this systematic review was to evaluate the content validity of Patient-Reported Outcome Measures (PROMs) that assess QoL in patients with a fistula. Methods: MEDLINE, EMBASE, PsycINFO, and Scopus were searched and studies assessing the content validity of patient-reported QoL measurement instruments, or PROM development studies in patients with cryptoglandular anal fistulae, were included. Data were extracted from eligible studies to determine the instruments’ relevance, comprehensiveness, and comprehensibility, and their quality was assessed according to COnsensus-based Standards for the Selection of health Measurement Instruments (COSMIN). Results: Two PROM development studies were identified, both of which described the development of a disease-specific QoL measurement instrument for patients with cryptoglandular anal fistulae. The overall content validity of these instruments was inconsistent and supported by very low-quality evidence. There were no studies assessing the content validity of established QoL measurement instruments in patients with fistulae. Conclusions: This systematic review could not establish the content validity of the available QoL PROMs for patients with anal fistulae, due either to the absence of designated content validity studies or a lack of comprehensiveness of the available PROMs. This highlights an important gap in the literature that needs to be addressed to ensure high-quality outcome assessment in patients with fistulae.

## 1. Introduction

An anal fistula is an epithelialised channel that links the inner surface of the anus or rectum with the external perianal skin. The majority of cases are cryptoglandular, occurring as a result of chronic suppurative infection, with Crohn’s disease, tuberculosis, and malignancy being less frequent causes [1,2]. Anal fistulae occur in approximately 1–2 people per 10,000/year in Europe [3] and, despite having been described in surgical literature for centuries, challenges in their treatment and the subsequent impact on patient quality of life (QoL) have endured. Patients typically experience perianal pain, discharge, and recurrent infective abscesses, all of which require significant adjustment of daily activities [4], making QoL improvement a key treatment goal. This is particularly true in complex and recurrent cases, where the ultimate aim of long-term healing is difficult to achieve. A recently developed Anal Fistula Core Outcome Set selected QoL as one of the most important outcomes that should be measured in all studies of fistula treatment [5]. Despite this, QoL is measured in only 14% of studies, using a range of measurement instruments [6].

QoL measurement is essential in chronic diseases where mortality is unaffected, as it can provide a more holistic and relevant assessment beyond that of physiological functioning and morbidity alone. However, the construct itself is not directly measurable and is largely subjective. Therefore, it is crucial that any measurement instrument used to assess QoL is valid, reliable, and relevant to the population within which it is used [7]. Patient-reported outcome measures (PROMs) are used to evaluate QoL in patients with fistulae [6]; however, their measurement properties have not yet been assessed in this patient population. Content validity, defined as the degree to which the content of an instrument reflects the construct to be measured in a particular patient population, is considered the most important measurement property for such tools and is determined by whether the items in the PROM are relevant, comprehensive, and comprehensible for that population. If a PROM fails adequately to capture the most salient aspects of a disease process, it may fail to demonstrate responsiveness, and the inclusion of irrelevant items can reduce its structural validity and internal consistency [7,8]. For a measurement instrument to be selected to assess an outcome within a Core Outcome Set (COS), it must at least have high-quality evidence of good content validity as determined by Consensus-based Standards for the selection of health Measurement INstruments (COSMIN) methodology [9].

The aim of this systematic review was to assess the content validity of measurement instruments used to assess Quality of Life (QoL) in patients with cryptoglandular anal fistulae using the COSMIN methodology [8].

## 2. Materials and Methods

This systematic review was conducted and reported according to the Preferred Reporting Items for Systematic Reviews and meta-analysis (PRISMA) guidelines. The COSMIN methodology for conducting a systematic review to critically appraise the measurement properties of PROMs was applied [7], and a protocol was registered in the international prospective register of systematic reviews (PROSPERO, registration number CRD42020204711).

### 2.1. Scope

This review evaluated PROMs assessing Quality of Life (QoL) in patients with cryptoglandular anal fistulae, to be used as outcome measurement instruments in clinical trials and clinical practice.

### 2.2. Search Strategy

MEDLINE, EMBASE, PsycINFO (via the Ovid interface), and Scopus were searched from inception to present on 6 September 2020, with an updated search performed in January 2022. A search strategy focusing on the four components of this review was constructed, including MeSH terms and key words for the following: (1) Quality of Life (construct), (2) patients with cryptoglandular anal fistulae (population), (3) Patient-Reported Outcome Measures (PROMs; type of instrument) and (4) measurement properties, including content validity. Comprehensive search filters were used to retrieve studies on the latter two components, which were adapted for each database [10]. In addition, we previously identified instruments commonly used to assess QoL in patients with cryptoglandular anal fistulae in contemporary research studies [6]. Additional searches were conducted using the names of these PROMs and ‘anal fistula’ search terms to identify studies satisfying the inclusion criteria specific to these instruments. Details of the search strategy are included in Supplementary File S1.

#### Study Selection

All study types were eligible for inclusion if they met three of the following criteria:

- The study aim was to assess the content validity of PROMs assessing QoL or
- Studies describing the development of patient-reported QoL measurement instruments
- More than 50% of the study sample had an anal fistula of cryptoglandular aetiology
- Published as a full-text article

Articles that were abstract only, studies of patients with Crohn’s anal fistulae, or studies that only used the PROM as an outcome measurement instrument were excluded. Two reviewers independently assessed all resulting abstracts (NI and RS) and potentially eligible studies were identified. Full-text reviews for these articles were conducted and any disagreement was resolved by the senior author.

### 2.3. Quality Assessment and Data Extraction

In accordance with COSMIN guidance [8], the quality of PROM development studies was determined against 35 items addressing the general design requirements of the study, the quality of methods used for concept elicitation, and the quality of any cognitive interviews or pilot tests conducted. The quality of content validity studies was determined by assessing each study against 31 items relating to whether patients were asked about the relevance, comprehensiveness, and comprehensibility and clinicians were asked about the relevance and comprehensiveness of the PROM depending on the available information. Each item was rated on a 4-point scale as very good, adequate, doubtful, or inadequate, and an overall rating for each PROM development or content validity study was obtained by taking the lowest rating of any item (“worse score counts” method).

**Figure 1:**
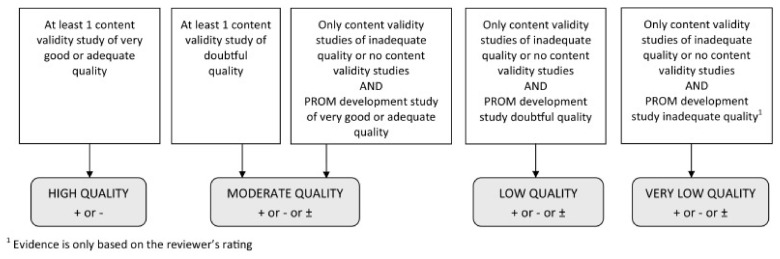
Modified GRADE approach for grading the quality of evidence. From Terwee et al. (2018) [8].

These assessments in addition to the reviewer’s own rating of the content of the PROM were assessed against the 10 criteria for good content validity as determined by COSMIN (8). These results were then qualitatively summarised to determine the PROM’s overall relevance, comprehensiveness, and comprehensibility, and overall content validity. Each criterion and the overall rating was determined to be sufficient (+), insufficient (−), inconsistent (+/−), or indeterminate (?) according to the results of evidence synthesis. Finally, the quality of evidence was determined using a modified GRADE approach, where the starting point is the assumption that the evidence is of high quality, which is then downgraded when risk of bias or inconsistency in results is identified (Figure 1). Two reviewers (NI, PT, or LA) independently appraised each eligible study and made judgements regarding quality of evidence and overall content validity, before coming together to discuss their ratings and achieving consensus on all items assessed.

## 3. Results

### Search Results

**Figure 2:**
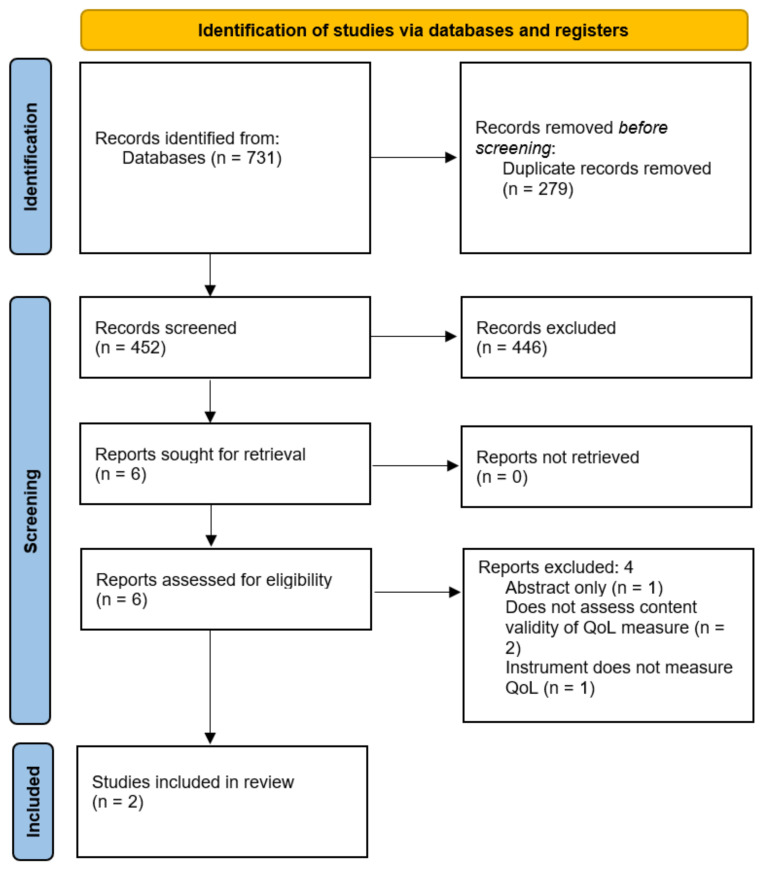
PRISMA diagram. QoL = Quality of Life.

A total of 731 studies were identified by the search, of which 452 underwent abstract screening following the removal of duplicates. The vast majority of these studies were deemed ineligible due to the use of PROMs in outcome assessment only, with very few assessing the measurement properties or validity of these instruments. Six studies were identified for potential inclusion, of which one was the abstract of a full-text publication that was included in the review [11], two described the correlation of QoL with other constructs such as patient satisfaction [12] and severity of faecal incontinence [13] in patients following fistula treatment, and a further study described the use of a PROM as an outcome measurement instrument [14]. As a result, two studies were eligible for inclusion in this review, both of which described the development of disease-specific QoL PROMs in patients with cryptoglandular anal fistulas [11,15] (Figure 2). There were no content validity studies conducted for any QoL PROMs in anal fistula patients.

The Perianal Sepsis Index (PASI) [11] was developed as an index that could be used by clinicians and researchers undertaking clinical trials to measure outcomes of treatment, focusing on QoL issues associated with cryptoglandular sepsis. It was developed by asking 20 non-Crohn’s-disease patients with active perianal sepsis (all of whom had diagnosed anal fistulae) for more than 3 months the five most significant problems they experienced due to ongoing sepsis affecting their QoL. The score consists of three items measured on a 5-point Likert scale, assessing pain and discomfort, discharge and leakage, and sexual function and limitation, mirroring the patient reported components of the Perianal Disease Activity Index [16].

The Quality of Life Anal Fistula Questionnaire (QoLAF-Q) [15] was developed to assess QoL in patients with an anal fistula, in order to aid decision making in treatment strategies. There are 14 items included in the final PROM, which was developed by researchers based on their experiences with patients, the most frequent clinical manifestations of anal fistulae, and the domains included in the World Health Organisation Quality of Life assessment, and refined by a panel of 14 expert coloproctologists assessing the content validity of each item. The final PROM then had its readability and understandability assessed by a sample of 10 independent native Spanish speakers and 5 non-native speakers, who were asked to comment on any difficulties found when reading and completing the questionnaire. Criterion validity and reliability were also assessed and Principal Component Analysis was performed [15].

The overall evaluation of PROM development for both instruments is shown in Table 1. Neither study used cognitive interviews or pilot testing. Therefore, this part of the assessment could not be completed.

Neither PROM development study provided a concise description of QoL as a construct, or its theoretical basis or boundaries. However, both provided broad descriptions of their target population as patients with a cryptoglandular fistula or sepsis with an evaluative context of use. There was a lack of detail provided on the use of any qualitative data collection or analysis methods; therefore, the majority of the concept elicitation criteria were rated as doubtful. For the QoLAF-Q, patients were not involved in concept elicitation; therefore, this part of the PROM development study could not be assessed.

Taking the lowest rating of any item, the total quality of PROM design for both instruments was deemed to be inadequate, and therefore the total quality of both PROM development studies was also rated as inadequate.

Although no specific content validity studies had been conducted for either instrument, the development of QoLAF-Q included testing the readability and understandability of the final instrument (Table 2). There was an absence of detail regarding the methodology (i.e., whether this was done through qualitative interviews) or whether participants were specifically asked about the comprehensibility of response options, instructions, and recall period. Therefore, the overall quality of the comprehensibility study was rated as doubtful. Clinicians were asked to rate the relevance of items on the QoLAF-Q; however, this was conducted as part of PROM development study, so this aspect cannot be assessed as a content validity study as per COSMIN guidance.

The overall ratings for content validity and quality of evidence can be found in Table 3. Given that the PROM development studies were deemed to be of inadequate quality, the overall ratings were determined by reviewer assessments, and therefore judged to be very low-quality evidence by COSMIN criteria (Figure 1). The reviewers agreed that although both PROMs contained items that were relevant to QoL in anal fistula patients, they lacked comprehensiveness and focused on only a few areas impacted by the disease. For this reason, an overall inconsistent rating was given for the content validity of these instruments. Based on these results, we cannot establish which extant PROM has the greatest content validity for QoL in anal fistula patients.

## 4. Discussion

Quality of Life has emerged as a crucial outcome to consider in the treatment of anal fistulae [5], making it essential to use measurement instruments that adequately reflect the profound impact that the disease and its treatment can have. A range of generic QoL measurement instruments have been used in fistula research. This systematic review using validated search filters was not able to identify any studies that assessed the measurement properties of these instruments in this patient group. Two disease-specific QoL PROM development studies were identified. However, the lack of construct definition, methodological detail, patient involvement in development, and cognitive interviews or pilot testing meant that the quality of these studies was inadequate according to COSMIN standards. This meant that overall content validity was determined by reviewer ratings. These were conducted by clinicians with experience in the management of patients with anal fistulae and in qualitative research, who suggested that whilst the items in both PROMs were relevant, they did not encapsulate the complexity of QoL in these patients.

Qualitative investigation into this patient group has identified the far-reaching effects of having a fistula, influencing work patterns and planning, relationships, daily activities, and psychological well-being [4]. Whilst the PROMs evaluated here should be lauded for attempting to address disease-specific aspects of QoL and including items known to be relevant to fistula patients, such as limitation of sexual function, some key aspects are missing, for instance, neither instrument asks about the impact on work or distinguishes how a seton, frequently used as long-term treatment, may positively or negatively impact QoL. Patient involvement in PROM development, using in-depth qualitative methods, may help to address the deficits in current measurement instruments.

Generic QoL measurement instruments are used in the study of many chronic diseases, such as stroke [17], diabetes [18], and heart disease [19]. In the study of anal fistulae, instruments such as the Short Form-36, Short Form-12 [20,21,22,23], the Cleveland Global Quality of Life score [24,25], and EQ 5D [26,27] have been used to assess QoL. Although these have the benefit of allowing comparison between diseases, patients with anal fistulae have described how QoL issues are tightly tied into their unique symptom profile [4,5], and so these generic instruments may not be sensitive to subtle changes in symptoms, particularly in response to treatment. Improvement in QoL is a highly valued outcome for those with complex disease where a complete cure is unlikely; therefore, it is important that selected QoL instruments can demonstrate sensitivity to change in these patients. They must also be able to detect specific negative effects on QoL due to interventions.

Symptom-specific instruments such as the Gastrointestinal Quality of Life Index (GIQLI) [28,29,30] and the Faecal Incontinence Quality of Life Scale (FIQLS) [27,31,32] have also been used in studies of fistula treatment. The GIQLI consists of 36 items addressing five domains of symptoms; physical, emotional, and social dysfunction, and effect of medical treatment. It was developed on patients with benign and malignant GI disorders including anorectal disease, but there are no specific details regarding their diagnoses. Items in the symptom domain focus on abdominal pain, bloating, and bowel movements [33], all of which are unrelated to cryptoglandular anal fistulae. Its use in patients with benign anorectal disorders has been studied, including a cohort of 22 fistula patients, finding that QoL as measured by the instrument did not differ significantly in fistula patients, those with haemorrhoids, or a symptomatic rectocele when compared to age-matched controls [34]. The study suggested that this could be explained by patient adaptation to symptoms. However, it could be argued that this finding is largely artefactual, since these patients would be unlikely to demonstrate poor QoL if it was measured using questions about symptoms that are largely irrelevant to their disease process. The FIQLS was developed by expert clinicians and researchers and pretested in patients with faecal incontinence, demonstrating convergent validity with SF-36 [35]. Although faecal incontinence is an important aspect of disease that adversely impacts QoL in fistula patients, it is not universally suffered by all and should theoretically be less of a risk in treatments aimed at sphincter and continence preservation. However, many patients describe the presence of a seton, a stoma, or the management of ongoing discharge as being more prominent determinants of QoL, and so to assess QoL using questions centred around ‘accidental bowel leakage’ may give an inaccurate and understated portrayal of disease impact. In order to determine whether pre-existing generic and symptom-specific measurement instruments can accurately assess QoL in this patient cohort, well-designed content validity studies are required, using content and lay experts ideally composed of clinicians familiar with, and, more importantly, patients suffering from the disease, to assess the relevance, comprehensiveness, and comprehensibility of the items contained within these instruments. Only then can we confidently assess which instrument is best suited to measure QoL in patients with cryptoglandular anal fistulae.

This study has its strength in using a broad search strategy with validated filters, implemented according to COSMIN guidance. However, it is limited in what it has been able to achieve, given the lack of studies that have assessed measurement properties of instruments used in this patient group. Despite this, we identified a gap in the literature that should be addressed in order to improve the assessment of these patients. Authors should discuss the results and how they can be interpreted from the perspectives of previous studies and of the working hypotheses. The findings and their implications should be discussed in the broadest context possible. Future research directions may also be highlighted.

## 5. Conclusions

Assessing the impact of fistula treatment on quality of life is a crucial part of fistula research; however, we have demonstrated that the validity of most measurement instruments that are selected for this use have not been adequately assessed for content validity. The few disease-specific QoL instruments available lack comprehensiveness. In order to improve the measurement of QoL in this patient group, content validity studies should be conducted on the instruments currently in use in the literature, and future PROM development should involve patient participants in concept elicitation and pilot testing.

## Figures and Tables

**Table 1:** Evaluation of PROM design according to COSMIN criteria for PROM development studies (COSMIN box 1a).

COSMIN Standards	Peri-Anal Sepsis Index (PASI) [8]	Quality of Life Anal Fistula Questionnaire (QoLAF-Q) [12]
**General design requirements**		
Is a clear description provided of the construct to be measured?	Inadequate	Inadequate
Is the origin of the construct clear?	Doubtful	Doubtful
Is a clear description provided of the target population for which the PROM was developed?	Very Good	Very Good
Is a clear description provided of the context of use?	Very Good	Very Good
Was the PROM development study performed in a sample representing the target population for which the PROM was developed?	Very Good	Inadequate
**Concept elicitation**		
Was an appropriate qualitative data collection method used to identify relevant items for a new PROM?	Doubtful	Not assessed
Were skilled group moderators/interviewers used?	Doubtful	
Were the group meetings or interviews based on an appropriate topic or interview guide?	Doubtful	
Were the group meetings or interviews recorded and transcribed verbatim?	Doubtful	
Was an appropriate approach used to analyse the data?	Doubtful	
Was at least part of the data coded independently?	Doubtful	
Was data collection continued until saturation was reached?	Doubtful	
For quantitative studies: was the sample size appropriate?	Inadequate/Not applicable	

COSMIN = Consensus based Standards for the selection of Measurement INstruments, PASI = Peri-Anal Sepsis In-dex, QoLAF-Q = Quality of Life Anal Fistula Questionnaire.

**Table 2:** Asking patients about the comprehensibility of the QoLAF-Q.

COSMIN Standards for Asking Patients about Comprehensibility of a PROM	Quality of Life Anal Fistula Questionnaire (QoLAF-Q) [12]
Was an appropriate qualitative method used for assessing the comprehensibility of the PROM instructions, items, response options, and recall period?	Doubtful
Was each item tested in an appropriate number of patients?	Very Good
Were skilled group moderators/interviewers used?	Doubtful
Were the group meetings or interviews based on an appropriate topic or interview guide?	Doubtful
Were the group meetings or interviews recorded and transcribed verbatim?	Doubtful
Was an appropriate approach used to analyse the data?	Doubtful
Were at least two researchers involved in the analysis?	Doubtful

COSMIN = Consensus based Standards for the selection of Measurement INstruments, QoLAF-Q = Quality of Life Anal Fistula Questionnaire.

**Table 3:** Overall ratings for content validity and quality of evidence.

PROM (Subscale)		PROM Development Study	Rating of Reviewers	OVERALL RATINGS PER PROM	QUALITY OF EVIDENCE	PROM Development Study	Content Validity Study	Rating of Reviewers	OVERALL RATINGS PER PROM	QUALITY OF EVIDENCE
		PASI				QoLAF-Q				
Relevance										
1	Are the included items relevant for the construct of interest?	?	+			?		+		
2	Are the included items relevant for the target population of interest?	+	+			+		+		
3	Are the included items relevant for the context of use of interest?	+	+			+		+		
4	Are the response options appropriate?	?	+			?		+		
5	Is the recall period appropriate?	?	?			?		?		
	RELEVANCE RATING (+/−/±/?)	?	+	+	Very low	?		+	+	Very low
Comprehensiveness										
6	Are all key concepts included?	?	-			?		-		
	COMPREHENSIVENESS RATING (+/−/±/?)	?	-	-	Very low	?		-	-	Very low
Comprehensibility										
7	Are the PROM instructions understood by the population of interest as intended?	?	+			?	?	-		
8	Are the PROM items and response options understood by the population of interest as intended?	?	+			?	?	+		
9	Are the PROM items appropriately worded?									
10	Do the response options match the question?									
	COMPREHENSIBILITY RATING (+/−/±/?)	?	+	+	Very low	?	?	+/−	+/−	Very low
	CONTENT VALIDITY RATING (+/−/±/?)	?	+/−	+/−	Very low	?	?	+/−	+/−	Very low

PROM = Patient Reported Outcome Measure, PASI = Peri-Anal Sepsis Activity Index, QoLAF-Q = Quality of Life Anal Fistula Questionnaire.

## Data Availability

Not applicable.

## Supplementary Materials

The following supporting information can be downloaded at: https://www.mdpi.com/article/10.3390/clinpract12040066/s1, Supplementary File S1: Search strategy.
